# Curriculum Mapping with Academic Analytics in Medical and Healthcare Education

**DOI:** 10.1371/journal.pone.0143748

**Published:** 2015-12-01

**Authors:** Martin Komenda, Martin Víta, Christos Vaitsis, Daniel Schwarz, Andrea Pokorná, Nabil Zary, Ladislav Dušek

**Affiliations:** 1 Institute of Biostatistics and Analyses, Faculty of Medicine, Masaryk University, Brno, Czech Republic; 2 Faculty of Informatics, Masaryk University, Brno, Czech Republic; 3 Department of Learning, Informatics Management and Ethics, Karolinska Institutet, Stockholm, Sweden; Penn State College of Medicine, UNITED STATES

## Abstract

**Background:**

No universal solution, based on an approved pedagogical approach, exists to parametrically describe, effectively manage, and clearly visualize a higher education institution’s curriculum, including tools for unveiling relationships inside curricular datasets.

**Objective:**

We aim to solve the issue of medical curriculum mapping to improve understanding of the complex structure and content of medical education programs. Our effort is based on the long-term development and implementation of an original web-based platform, which supports an outcomes-based approach to medical and healthcare education and is suitable for repeated updates and adoption to curriculum innovations.

**Methods:**

We adopted data exploration and visualization approaches in the context of medical curriculum innovations in higher education institutions domain. We have developed a robust platform, covering detailed formal metadata specifications down to the level of learning units, interconnections, and learning outcomes, in accordance with Bloom’s taxonomy and direct links to a particular biomedical nomenclature. Furthermore, we used selected modeling techniques and data mining methods to generate academic analytics reports from medical curriculum mapping datasets.

**Results:**

We present a solution that allows users to effectively optimize a curriculum structure that is described with appropriate metadata, such as course attributes, learning units and outcomes, a standardized vocabulary nomenclature, and a tree structure of essential terms. We present a case study implementation that includes effective support for curriculum reengineering efforts of academics through a comprehensive overview of the General Medicine study program. Moreover, we introduce deep content analysis of a dataset that was captured with the use of the curriculum mapping platform; this may assist in detecting any potentially problematic areas, and hence it may help to construct a comprehensive overview for the subsequent global in-depth medical curriculum inspection.

**Conclusions:**

We have proposed, developed, and implemented an original framework for medical and healthcare curriculum innovations and harmonization, including: planning model, mapping model, and selected academic analytics extracted with the use of data mining.

## Introduction

A correctly compiled and balanced curriculum that covers all theoretical and clinical fields of medicine is an essential prerequisite for medical doctor education [1]. The keyword “innovation” is perceived as an activity that enables higher education institutions (HEIs) to make their curricula more up-to-date, while maintaining transparency and a high degree of structure. The innovations, if performed in teaching domains formalized with the use of an unambiguous parametric description, and with entities adopted from the outcomes-based concept of education (LUs: learning units, LOs: learning outcomes), will enhance the transparency and continuity of the environment in which both teachers and students work daily [2,3]. In this manner, an innovation is more likely to have positive and practically applicable effects on higher medical education, if it is based on a balanced and well-structured medical curriculum.

Such efforts have recently demonstrated how a medical curriculum can be used as a tool to drive innovation for the sake of understanding and perceiving the complexity of medical education. This ability has proved that it is possible to verify the existence of the constructive alignment within the medical curriculum [4], and that performing a high-level gap analysis of the relationships between major curriculum components such as LOs, teaching activities, educational material, and assessment methods may eventually support critical decisions concerning quality improvement in the medical education. Different approaches like visual analytics [5–8], web-based learning objective databases [9], simple visualizations of academic analytics applied to a medical curriculum [10], and the results of our previous work [1,11–14], have all been used for this purpose. The aim of those studies was to improve the traditional method for aligning the medical curriculum and to strengthen the complicated decision making process, which is usually performed by medical education stakeholders such as teachers, tutors, curriculum designers, or institutional managers. That goal has previously been successfully if only partially addressed, as these studies investigated possible novel solutions at an exploratory level. A solution concerning the medical curriculum as a whole, which could enable its effective encapsulation, description, and management together with ability to demonstrate its most important aspects, is still missing.

### 1.1 Background of curriculum mapping

The advent of information technology in various educational domains has led to large volumes of data that is stored in various formats, including students’ data, teachers’ data, alumni data, resource data, curriculum data, etc. A multidisciplinary area called Knowledge Discovery in Databases (KDD) covers several methodologies for extracting useful information from data, including database design, statistics, pattern recognition, machine learning, and data visualization. A set of proper KDD methods is required to uncover knowledge from these large data repositories, in order to improve understanding and decision making [15]. By adopting these proven methodologies for extracting useful information from the large databases of curriculum management systems, we are able to create visual representations of the curricula that are based on real time information; this is one way to increase collaboration and collegiality in HEIs. This technique is called curriculum mapping. It introduces two main objectives: (i) to make the curriculum more transparent to all stakeholders; and (ii) to demonstrate the links and relationships between the various components of the curriculum. In general, curriculum maps can identify whether the intended material is actually being taught and what students actually learn, and demonstrate the links among the different components of the curriculum. The key to a really effective integrated curriculum is to get teachers to exchange information about what is being taught and to coordinate this so that it reflects the overall goals of the institution [16–19]. Curriculum mapping is about spatially representing the different components of the curriculum so that the whole picture and the relationships and connections between the parts of the map are easily seen. There are several case studies that have been successfully implemented in practice [5,20–24]. One of the main objectives of this paper is to introduce the use of mapping for the effective evaluation of medical curriculum, and to provide automatic tasks that could be used to build well-balanced courses that are both theoretically- focused and clinically-based.

## Methods

### 2.1 Planning model for curriculum innovations

**Fig 1:**
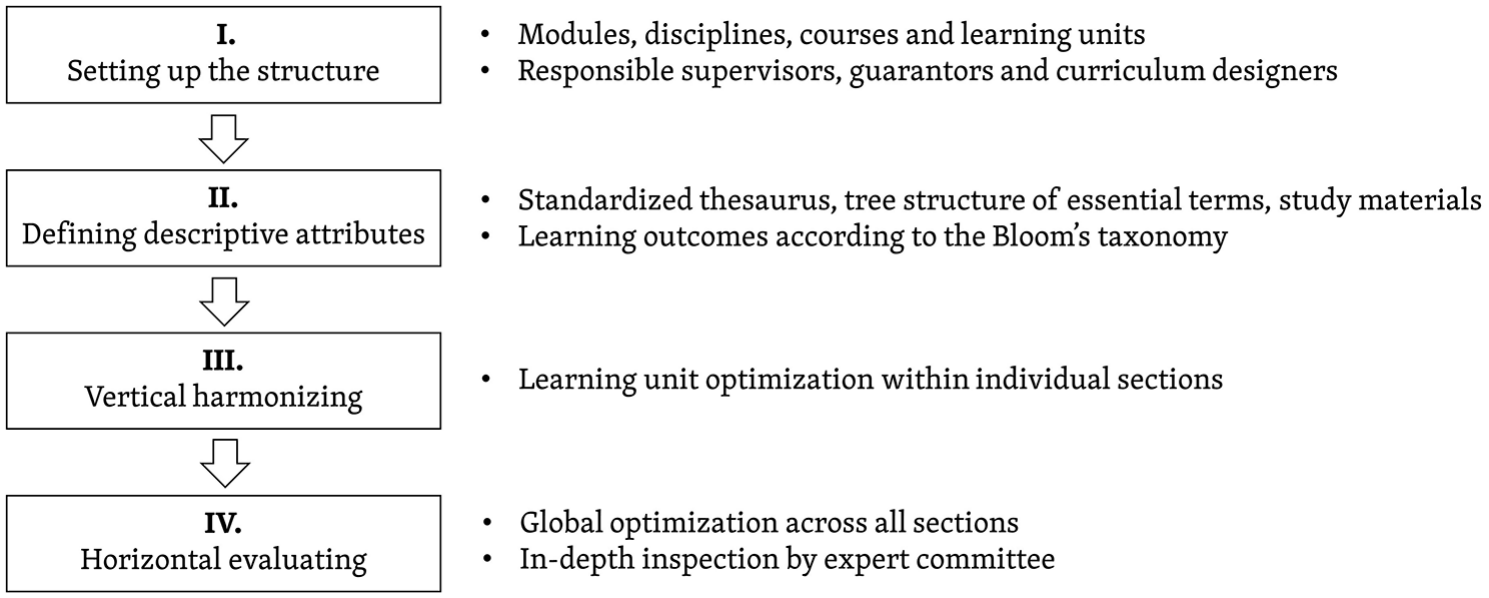
The planning model introducing innovations into a curriculum.

Our research is concentrated on proposing a way to channel clear communication between stakeholders (i.e., curriculum designers, supervisors, guarantors, faculty management, and students). On the grounds of the long-term and iterative form of curriculum innovations, we have already designed and implemented an in-house curriculum management system [25], which offers a wide range of web-based tools suitable to implement the innovations efficiently. The curriculum planning model (see Fig 1) that is supported by the curriculum management system helps academics reengineer their curriculum activities. The planning process is delineated in three major stages and is followed by the final evaluation process. (i) Setting up the medical curriculum structure. The study field is divided into individual sections, medical disciplines, courses, and LUs, including the responsible supervisors and guarantors. (ii) Defining the descriptive attributes of the LUs, linked MeSH vocabulary keywords and essential terms, and associated Los, according to Bloom’s taxonomy. The outcomes typically consist of a noun or noun phrase (i.e., the subject matter content) and a verb or verb phrase (i.e. the cognitive process). In this case, each LO defines what students are expected to know, understand, and/or be able to demonstrate at the end of the learning period, typically as a graduate. This concept has been already applied by a number of academic institutions, especially in medical education [26,27]. (iii) Vertical harmonizing, i.e., LU optimization and further discussion within individual sections, under the supervision of responsible guarantors. (iv) Horizontal evaluating, i.e., follow-up discussions across all sections under the management of supervisors, including an in-depth inspection in collaboration with an established expert committee, which can logically influence the whole structure of the defined curricula. The model represents an original methodology for the creation of a new structured set of LOs designed to assess and adjust to real education.

### 2.2 Curriculum mapping model

**Fig 2:**
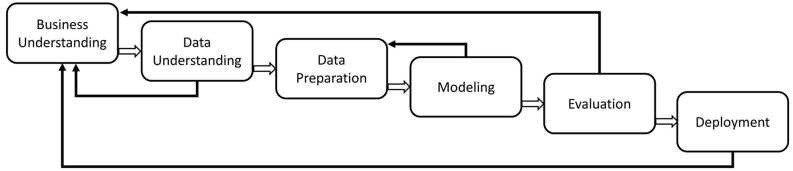
The six phases of the CRISP-DM reference model [30].

Following the formal description of the medical curriculum [28], we decided to use selected KDD methods to uncover novel and potentially useful information from the medical curriculum data [29]. To increase the success and accuracy of solving individual KDD tasks, the CRISP-DM (Cross-Industry Standard Process for Data Mining) methodology was chosen. It defines a non-rigid sequence of six phases, which allow researchers to build and implement various data mining models to be used in a real environment (see Fig 2).

From an informatics perspective, pre-processing and analysis of available data stored in a curriculum management system is challenging. We have already prepared the normalized database schema based on the formal description of curriculum, which enables easy extraction of information. We have identified many KDD tasks in a form advanced analysis of medical curriculum, which can significantly help curriculum designers with the construction and maintenance of a well-balanced curriculum. These are selected questions that the application of KDD algorithms may help to answer:

- Which educational domains (medical disciplines) do not conform to an expected form within the curriculum data?
- Can we identify the most outlying areas and undesirable overlaps across the medical curriculum?
- Which communities/clusters of medical disciplines are we able to identify in the curriculum?
- Which courses belong to the most important parts of the curriculum (with respect to “content distance” to other courses)?

The proposed set of steps, in accordance with the six-stage-sequence of the CRISP-DM process, facilitates the search for potentially problematic areas and the construction of comprehensive reports for subsequent global, in-depth inspection.

#### 2.2.1 Business understanding

This initial phase is focused on understanding the objectives and problem definition in terms of medical curriculum innovation. The goal is to detect outlying and overlapping areas in the General Medicine study program, using data/text mining techniques. The results of the data mining might then be the subject of an evaluation provided by senior curriculum experts. The mentioned goal can be reformulated in terms of data mining issues, such that the task is to investigate the properties of a graph obtained from a dissimilarity matrix over a collection of documents representing individual parts of the curriculum. Our approach is based on Social Network Analysis (SNA), and it was chosen since it can naturally deal with graph-like/network-like data and encompasses centrality notions. First of all, we generate the entire similarity graph based on the similarities of individual medical disciplines as represented by their textual descriptions; specifically, the cosine distance is used. Next, we compute various centrality measures to model the interconnection and remoteness of the nodes (i.e., medical disciplines, in our particular case) in the graph. We are interested in uncovering densely connected subgraphs called communities, as these might represent the most crucial and important parts of a curriculum, in terms of its global harmonization. Based on the community detection, we are able to explore novel and useful information about the structure of the General Medicine study program. We can identify the nodes with extreme (i.e., the highest/the lowest) values of selected centrality measures, which may indicate that these medical disciplines belong to an essential part of the curriculum, or, conversely, it may cause undesirable preference of mentioned disciplines that in fact cannot be identified by manual human inspection. A similar approach has been proposed by Trigo and Brazdil on mining affinity relationships within groups of researchers [31].

#### 2.2.2 Data understanding

**Fig 3:**
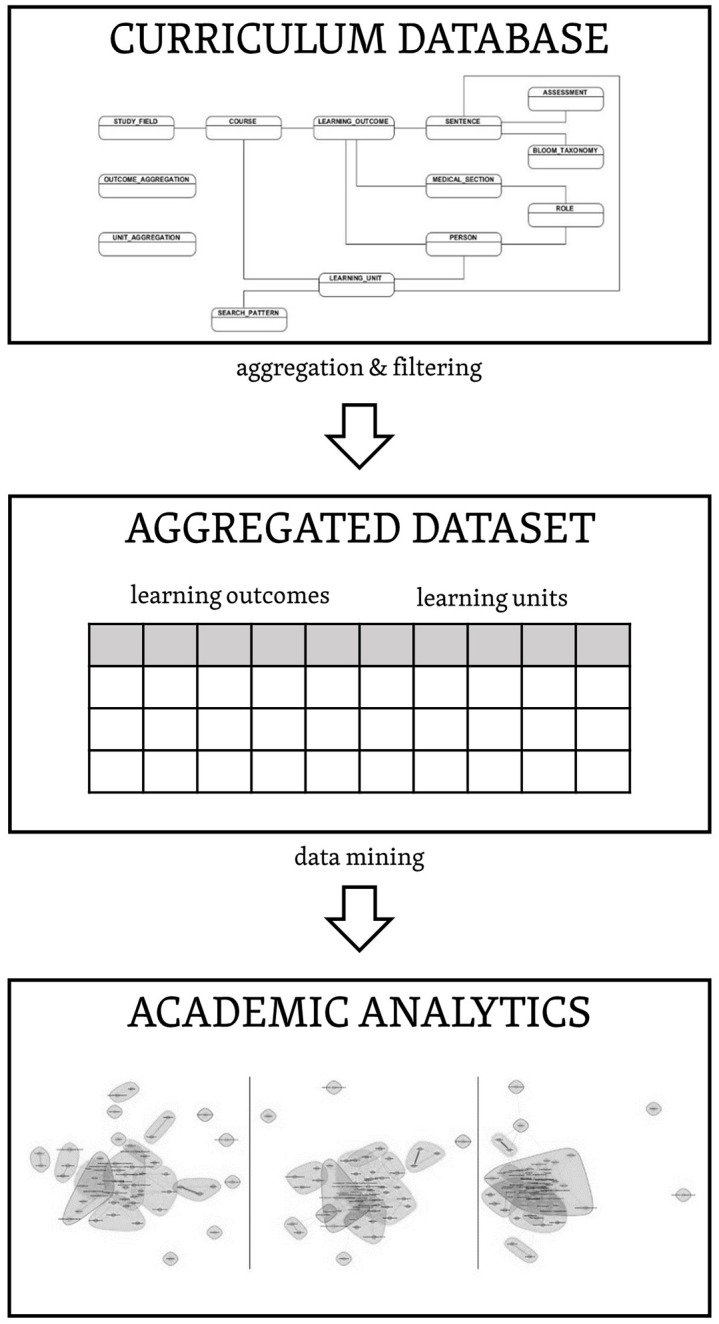
Phases of the CRISP-DM reference model.

We have already built the database for collecting medical curriculum data in a parametric format. It covers all elements pertaining to global curriculum harmonization, including detailed metadata specification down to the level of LUs, linkages to Los, and standardized biomedical vocabulary. The organization of the metadata and its linkages is provided in the curriculum model, which can be implemented without any restrictions within any relational database technology. In order to effectively process curricular metadata stored in our system, we used aggregation and filtering to get the final dataset from the primary data, followed by application of particular KDD methods for exploring new knowledge (see Fig 3).

#### 2.2.3 Data preparation

This step represents an important preprocessing step, which then allows the use of any of the data mining algorithms. All the stated content was created in the Czech and English languages. For the purpose of in-depth analysis of the present pilot, the English version was used, due to easier preprocessing. We identified a set of information-rich attributes, in collaboration with curriculum designers and guarantors, which are suitable for exploring the medical curricula. In our case, for each discipline a single plaintext file was generated, so that it contained merged contents of several attributes (see Table 1) describing LO and its affiliation to curriculum (i.e. link to LU, course, medical discipline, and section). For the purpose of analytical pilots, we processed only the attributes that were related to the particular LOs (i.e., sentences with action verbs, according to Bloom taxonomy) and to all of the descriptive indexes (i.e., grouped outcome, primary index, secondary index). For illustration, the input dataset could have been quantified by approximately 636 normative pages (containing 1,800 characters of text) or with 118,798 words.

**Table 1:** Attributes of final dataset describing the medical curriculum.

Attribute	Sample
**LO**	
Group outcome	Hypothalamic-pituitary portal system, liberins, statins
Primary index	Endocrinology
Secondary index	Hypothalamus
Sentence	Student is able to assign functional characteristics of different hormones to the structure of the hypothalamic-pituitary system.
Assessment form	Final exam—oral
**LU**	
Name	Endocrine system
Courses	Physiology I—practice, Physiology I—lecture
Medical discipline	Physiology
Section	Theoretical sciences
Importance	The aim of this teaching unit is to introduce students to the basics of the physiological functions of the endocrine system. The students will be able to compare basic types of intercellular communication (endocrine, paracrine, autocrine).….
Description	The purpose of this teaching unit is to introduce students to the basics of endocrine control of the body. First, it broadly defines the general principles of regulation and the role of the chemical structure of signaling molecules in the pathway of effector response activation…
MeSH keywords	Insulin, Endocrine gland
Significant terms	Glandula suprarenalis, Facies, Hilus, Pancreas, …

#### 2.2.4 Modeling

This phase uses modeling techniques (i.e., analytic methods, particularly data mining methods) on the dataset prepared in the previous phase. The entire text preprocessing was implemented with the use of the R software and its standard packages tm (a widely used package for text mining that allows us to deal with collections of textual data in a convenient way) and lsa (a standard package for latent semantic analysis). The collection of plaintext files that represent individual disciplines was loaded as a corpus (from tm package) in order to prepare their bag-of-word representation. A usual sequence of pre-processing issues was applied for each document in the corpus after tokenization: transformation to lowercase, stemming (using the Snowball system), punctuation removal, numbers removal, stop words removal, and whitespace stripping. The text preprocessing step was followed by the computation of two matrices. First, a document-term-matrix (DTM) based on *tf-idf* weighting [32] was generated. Consequently, a dissimilarity matrix was computed on the base of the cosine similarity over the vectors in the DTM. Values were rounded to two decimal digits, and values lower than a certain threshold were replaced by zeros, since extremely low similarities were considered irrelevant. This dissimilarity matrix naturally corresponds to an undirected graph with weighted edges. The dissimilarity matrix can be viewed as an adjacency matrix of this graph (where the nodes are the disciplines, the edges represent similarities between pairs of documents, and the weight of an edge corresponds with the similarity value); thus, we are able to use various SNA techniques to work with graph representation of data.

In our case study, we have concentrated on the problem of community finding and estimating the importance of each node by various centrality measures, which is an easy way to explore the real composition and intersections of individual disciplines in the curriculum. For community finding, the WalkTrap algorithm [33] was chosen, due to its low computing complexity and the fact that it is implemented within the igraph package. The algorithm is based on short random walks in the graph. Its main premise is that short random walks tend to stay in the same community [34]. Here, we experimented with different values of WalkTrap parameters, mainly with the length of the random walk. After uncovering the communities, the following three centrality measures were computed: closeness centrality, betweenness centrality, and Eigenvector centrality. The closeness centrality of a node is defined by the inverse of the average length of the shortest paths to all of the remaining nodes in the given graph. The (vertex/node) betweenness centrality is, in the simplest case, defined as the number of shortest paths that go through a given node. Eigenvector centrality is one way of computing the approximate importance of a given node. The idea behind this measure is that the centrality of each node is the sum of the centrality values of its neighboring nodes. More precisely, the Eigenvector centrality values correspond to the values of the first eigenvector of the adjacency matrix. In all cases, we will use versions of these measures for graphs with weighted edges, and all values are normalized. The short description of these measures is given below, and more details can be found in [35,36].

Closeness centrality: The closeness centrality of a node *v* in a graph *G* is defined by the inverse of the sum of the lengths of the shortest paths to/from all the other nodes in the graph *G*:

$$c\left(v\right)=\frac{1}{\sum_{i\in V\;\left(G\right),\;i\neq v}d\left(i,v\right)},$$ (1)

Where *d(i*, *v)* is the length of the shortest path from node *i* to node *v*. If there is no path between a pair of nodes, then the total number of nodes in the graph is used instead of the path length. Through the mentioned calculation, we obtain the so-called raw closeness of the node. To obtain the normalized closeness of the node *v*, we multiply the raw closeness by (*n − 1*), where *|V(G)| = n*, and we use this normalized version in subsequent calculations. Nodes (disciplines) with low values of closeness are those disciplines whose content is distant from other ones; thus, roughly said, they are independent of the others.

Betweenness centrality: In the simplest case (i.e., without edge weighting), the raw betweenness centrality of a node *v* corresponds with the number of shortest paths from all nodes to all others that go through the node in question:

$$b\left(v\right)=\;\sum_{i,j,\;v\;\in V\;\left(G\right),\;i\neq j,\;i\neq v,\;j\neq v}\frac{g_{ivj}}{g_{ij}},$$ (2)

Where *g*_*ij*_ is the total number of shortest paths going from node *i* to *j*, and *g*_*ivj*_ is the total number of all the shortest paths from node *i* to node *j* going through *v*. To get the normalized betweenness *b*_*n*_ *(v)* of the node *v*, we calculate $b_{n}\left(v\right)=\frac{2b\left(v\right)}{\left(n-1\right)\left(n-2\right)}$, where |V(G)| =n. This definition can be extended for weighted networks. The nodes (disciplines) with high betweenness centrality are the ones that are the best for joining the students’ knowledge from different collections of disciplines (i.e., through intersubject transfer of knowledge).

Eigenvector centrality: Eigenvector centrality is one of the methods of computing approximate importance, and is a measure of the influence of a given node. The idea behind this measure is that the centrality of each node is the sum of the centrality values of its neighbor nodes. More preciously, the eigenvector centrality values correspond to the values of the first eigenvector of the adjacency matrix. The eigenvector centrality, in our case, models identification of important disciplines in the curriculum. All three of these measures were implemented within the igraph package.

#### 2.2.5 Evaluation

In this phase, a checking procedure was performed in order to assess whether the approach we used was appropriate, in terms of its usability in real curriculum management. The obtained results were organized and presented in a clear way for the curriculum designers and the committee experts. From the perspective of medical teachers, who are often authors of LUs and LOs, several clear benefits were identified: (i) transparent overview over the medical education in a particular HEI; (ii) detection of any outlying LUs; and (iii) continuous monitoring of the consistency of a curriculum in the never-ending process of innovation. An example evaluation of the achieved analytical results obtained from senior curriculum experts is given in 3.1.2.

#### 2.2.6 Deployment

Creation of the models is generally not the end of the project. The knowledge that was gained needs to be organized and presented in a way that the academics can use on a long-term basis. It often involves applying live models within a HEI’s decision making processes. Depending on the requirements, the deployment phase can be as simple as generating an analytical report or as complex as implementing a repeatable data mining process across the HEI. We have systematically tailored our generic process model, in accordance with a pre-defined context of medical curriculum exploration. In general, we aim to analyze and consolidate the experiences of this single pilot project towards a follow-up mapping issues in form advanced analysis of medical curriculum for future usage in comparable contexts. Basically, a global strategy for mapping consists of five steps: (i) analyze a specific field of study; (ii) remove any details not applicable to given field; (iii) add any details specific to given field; (iv) specialize (or instantiate) generic contents according to concrete characteristics of given field; and (v) possibly rename generic content to provide more explicit meaning in a given context, for the sake of clarity [37].

## Results

### 3.1 Curriculum innovations in practice

**Fig 4:**
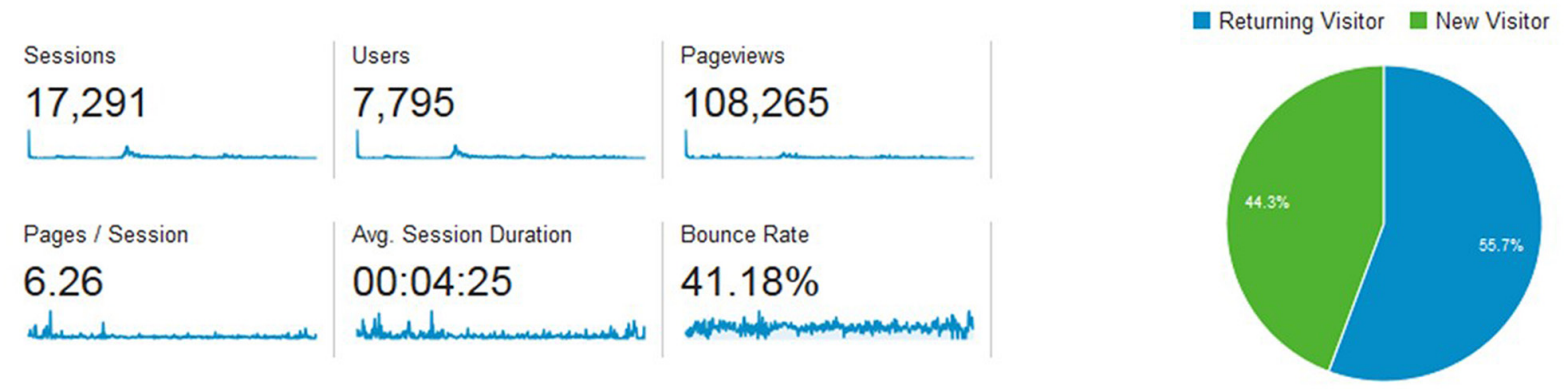
Google Analytics global overview (01 April 2014–01 August 2015).

**Fig 5:**
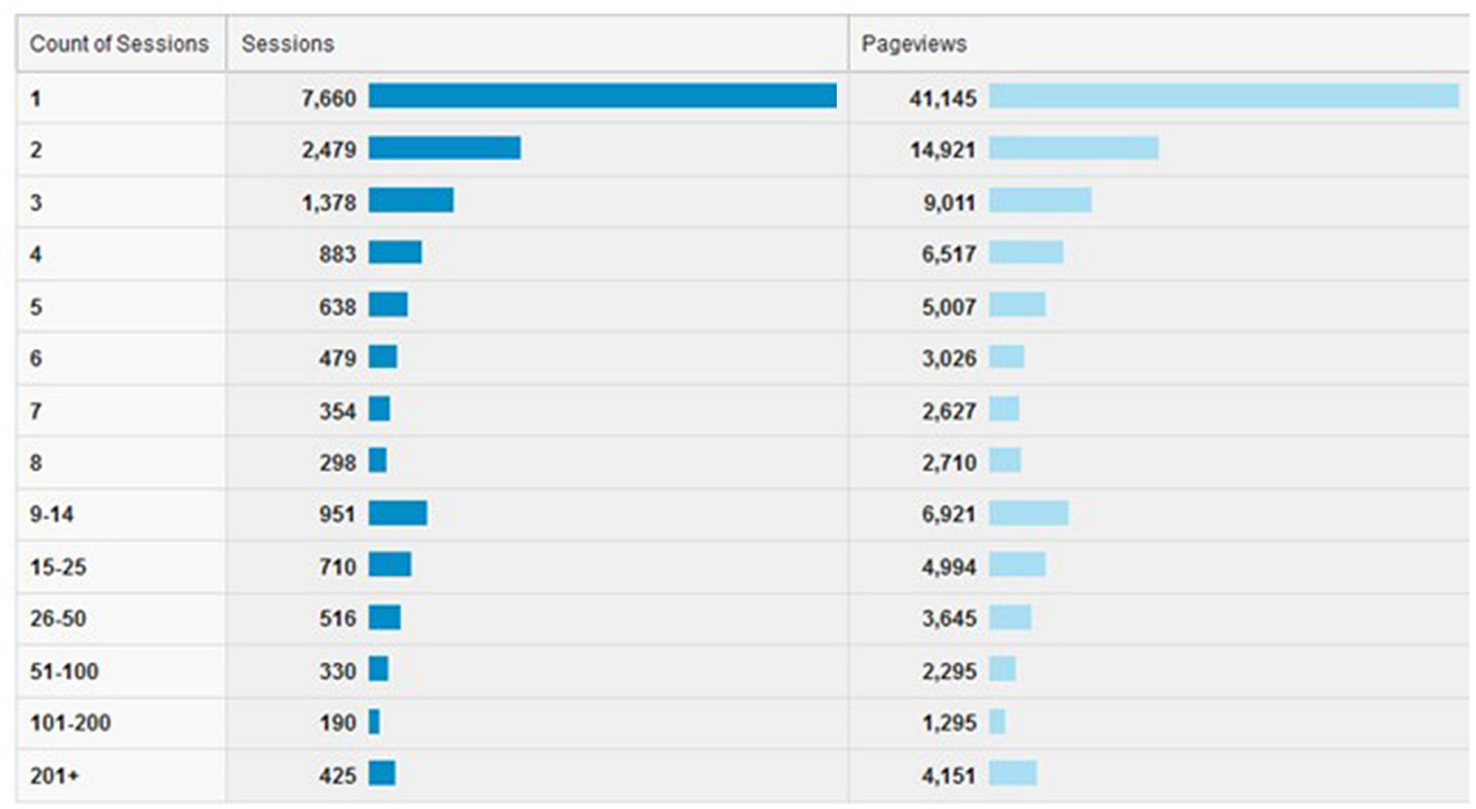
Google Analytics detailed overview of sessions (01 April 2014–01 August 2015).

The developed curriculum management system and relating planning model have been implemented into the practice in the Optimization of Medical Education (OPTIMED) project by the Faculty of Medicine of Masaryk University. Since 01 April 2014, OPTIMED has been fully open to all medical students and teachers. Depending on the various user roles, academics are able to use individual modules for global curriculum overview and also for managing the LUs and related LOs. Google Analytics monitoring was integrated into the system, thus up-to-date statistics, reports, and analyses based on site traffic and visitor's behavior is available. Figs 4 and 5 demonstrate the latest Google Analytics summary from 01 April 2014 to 01 August 2015, where audience behavior is reported.

Our approach to curriculum innovation offers an appealing way to effectively reform medical education, where the emphasis is on the product—what sort of graduates should be produced—rather than on the educational process itself. The primary effort in this project was a comprehensive curriculum innovation of the General Medicine study program. The innovations are driven towards a smoother continuity between the theoretical and clinical phases of the study, and by need to deliver graduates with 21st century skills. The key point of the project is the use of information and communication tools in order to achieve a horizontally-innovated structure of compulsory and compulsory-optional subjects. The objective is not a radical change in learning or teaching, but rather it is an exploratory mapping of the current state of the general medicine curriculum, with a prospect for innovations that produce more transparent educational environment [28]. The institutional management designated curriculum experts across medical disciplines, which interacted with the study harmonization and streamlining process in different roles (i.e., curriculum designers, guarantors, coordinators, reviewers). They proposed a set of fundamental knowledge and skills known as the Global Minimum Essential Requirements (GMER), in accordance with the proposed curriculum planning model, which is based on the outcomes-based paradigm. A total of 385 teachers have been interacting with the study harmonization and streamlining process for two years. Together, they have produced and evaluated the huge amount of metadata records that define the General Medicine study program, which are categorized to a pre-defined structure of medical curriculum (for illustration, it takes more than 2,500 normative pages of text).

The entire study field (General Medicine) is split into four individual sections (i.e., diagnostic and neurosciences, internal medicine, theoretical sciences, and surgical sciences), and includes details about the responsible supervisors. Each section contains a set of courses, which are divided into particular LUs. This categorization provides easy organization of the metadata about the education. Each LU covers LOs, which represent the basic requirements on the graduate from the selected field. All the curriculum metadata were collected using specialized modules within the developed platform, called LO & LU registers. They serve as online tools for medical curriculum management and provide detailed metadata specifications, down to the level of LUs and connections to the LOs. Table 2 gives the complete summary in numbers. In accordance with the proposed planning model, the vertical harmonization represents the verification and further discussion within the individual section, under supervision of the responsible guarantor. Finally, the horizontal evaluating brings the process of horizontal harmonization, which consists of follow-up discussions across all sections under the management of supervisors, including in-depth inspection in collaboration with an established expert committee.

**Table 2:** Summary of the descriptive attributes defining the medical curriculum.

Medical curriculum domain	Total number
Sections	4
Medical disciplines	44
Courses	144
LUs	1,347
LOs	6,974
Curriculum experts	385
Students	over 2,000

### 3.2 Curriculum mapping in practice

**Fig 6:**
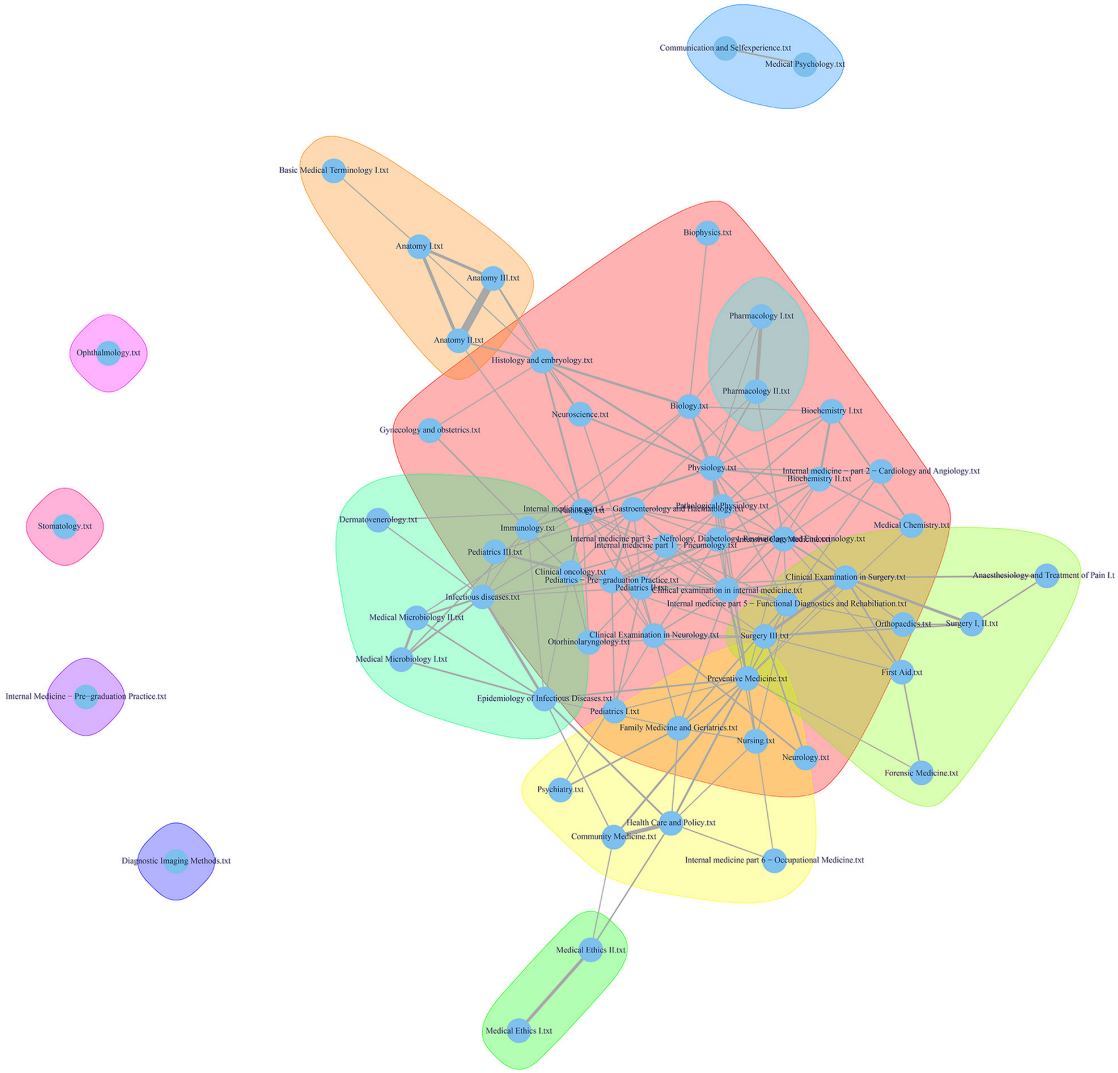
Overview of the medical curriculum, labelled with the communities that were uncovered using the Walktrap algorithm.

In accordance with CRISP-DM, we used the methods described above for solving the issue of medical curriculum mapping, which will enable medical curriculum designers and managers to better understand the multidimensional structure and complex content of the General Medicine study program. By applying the Walktrap algorithm to the collection of 59 documents (medical disciplines), we obtained a similarity graph, labelled with various colors, that defined a number of communities (Fig 6). The presented graph was computed with the length of the random walk set to *k* = 4; this setting provided the best results in terms of expert evaluation.

**Fig 7:**
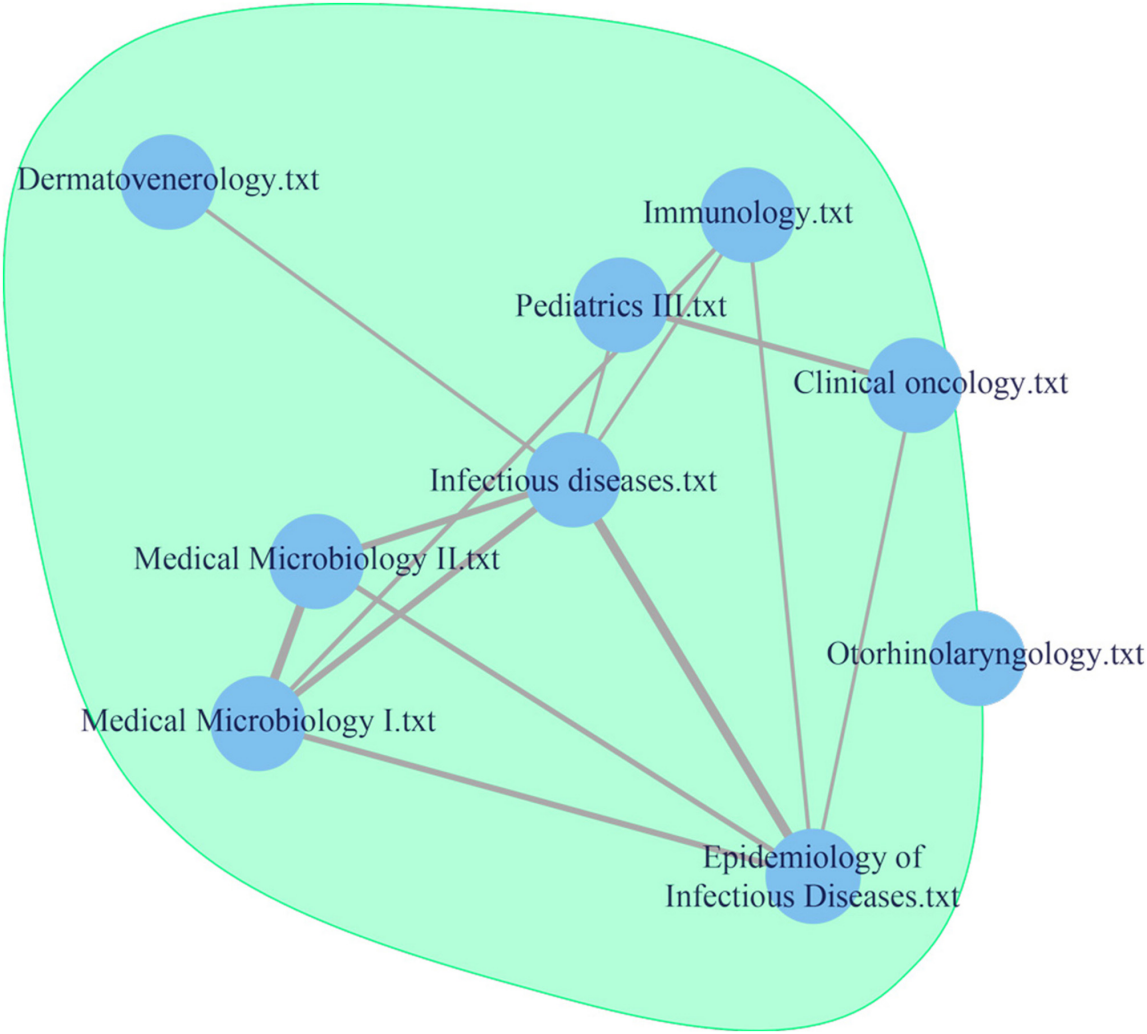
Detailed view of the interconnection between Microbiology and Infectious Diseases in different specialties.

To obtain an overall insight into the curriculum, the three centrality measures described in section 2.2.4 were used. Table 3 presents values of the centrality measures in alphabetical order for one particular community, which is shown as the enlarged curriculum map in Fig 7. Extremely low centrality values can indicate an inappropriate description of the discipline (e.g., missing important parts of the description). On the other side, the higher or extremely high values of the proposed measures can identify the core and most important parts of the curriculum.

**Table 3:** The values of betweenness, closeness, and Eigenvector centrality for one particular subset within the analyzed medical curriculum.

Medical discipline	Closeness centrality	Betweenness centrality	Eigenvector centrality
Clinical oncology	0.3084	0.1041	0.7991
Dermatovenerology	0.3042	0.0019	0.2070
Epidemiology of Infectious Diseases	0.3072	0.0309	0.6931
Immunology	0.3037	0.0047	0.3995
Infectious diseases	0.3071	0.0232	0.6795
Medical Microbiology I.	0.2975	0.0000	0.2588
Medical Microbiology II.	0.3001	0.0000	0.2400
Otorhinolaryngology	0.3052	0.0016	0.3744
Pediatrics III.	0.3048	0.0037	0.4866

## Discussion

### 4.1 Principal results

#### 4.1.1 Medical curriculum innovations

The Bergen ministerial conference of the Bologna Process in May 2005 discussed reforms to degree structures, credit transfer, quality assurance, and curricular development, which are transforming the European Higher Education Area. An outcomes-based approach is arguably best viewed as a fundamental building block of the Bologna education reforms, and brings greater transparency to higher education systems [38]. The process of medical curriculum innovation supported by a robust planning model, which is described here, is based on the well-established concept of a standardized definition of learning outcomes. One of its key features is its dynamism, namely the ability to easily manage any domain closely related to the medical curriculum through upgrades, and to absorb and incorporate all changes into the educational process. For teachers and faculty management, such an approach enables them to effectively administer education processes, to clearly see who teaches what and to what extent, to determine whether the lectures of individual teachers overlap thematically, to assess whether the overall teaching schedule is appropriate, or to decide whether a certain reform of the teaching schedule would be convenient and helpful. And for school managers, the presented tools will provide a practical view of the real content teaching. Further, it will also provide clear and comprehensible data about who teaches what and in what context, as well as information on the deficiencies and overlaps present in the curriculum. It is currently used by hundreds of senior teachers, curriculum designers, and professional guarantors, who use it for content definition and further inspection of the medical curriculum.

#### 4.1.2 Medical curriculum mapping

With respect to the achieved results, we decided to evaluate the curriculum in co-operation with senior guarantors (senior experts) of medical education at our university. The checking procedure is a mandatory phase of the CRISP-DM methodology, in order to make sure that the given objectives are fulfilled. Expert opinion helps to indicate whether the medical disciplines are balanced or unbalanced, and to identify weak points and shortcomings in terms of inconvenient interdisciplinary relations. In total, three experts were chosen, based on specific criteria: at least 10 years of experience in medical education (educational and scientific background), at least 15 years of professional clinical experience (following the award of the diploma), hold a managerial position in the medical faculty at a scientific level (i.e., at minimum, associate professor level), proven professional experience at a strategic level in medical education, thorough knowledge and proven experience in the principles of developing, applying, and implementing curriculum in medical education, and experience in managing educational projects/program concerning medical pre gradual education. All senior guarantors were familiar with the project activities, but were not deeply involved in the technical evaluation of the curriculum. All of them evaluated the generated graphs (see Figs 6 and 7) of the curriculum, and reviewed the pictures that described possible interconnections among each study subjects independently. The consensus of their review, based on their negotiations and discussion, is presented below. The reviewers were already familiar with the description of the technical evaluation process for evaluating interconnections (in terms of similarities, according the key words mentioned in the curriculum), and were introduced to some technical aspects of the evaluation, but without any guidance, or suggestions, or evocative recommendations, as this could influence the results of their review. As mentioned above, all reviewers held very similar positions, a fact that was due to set criteria mentioned above, and their views did not differ. All reviewers used a simple template to provide feedback, namely a checklist that evaluated the structure of the visualized curriculum. In addition, they described an evaluation in their own words, and discussed it afterwards all three experts together during an unstructured interview. This the method was used to analyze the qualitative feedback (simple semantic analyses and subsequent synthesis of conclusions was done).

Below, the short evaluation of similarity graphs is stated. Below, the short evaluation (a summary of comments) of similarity graphs is stated. The colored groupings of individual communities with related contents across the curriculum makes viewing the particular medical curriculum simpler and easier to understand. The courses that fall into the preclinical disciplines show suitable overlap and are centered together. The interconnection among the surgical disciplines, Anesthesiology, and First aid is excellent, implying good consistency. The distant location of the Ethics course is logical. However, this course is not interconnected with either Communication or Psychology; instead, it stands on the opposite side of the curriculum. It is possible that Ethics, Communication, and Psychology are not sufficiently represented in the preclinical and clinical disciplines, which unfortunately is a prevalent trend in medical education at Czech medical faculties [39]. Another explanation might lie in their different locations within the curriculum, in terms of the year of study (i.e., Ethics is taught in the second year of study). It is very difficult to interconnect specific ethical cases on which the education of ethics should be based, if students have not yet participated in clinical practice or internships. This problem might be addressed by extending the education of ethics to include the internship period.

An interconnection between Microbiology and Infectious Diseases (see Fig 5) can also be rationally explained: this confirms clear interdisciplinary relations. In this context, Dermatology and Venereology are also suitably located, because they can be regarded as specialties focused on the most common infectious diseases. the distant location of the Stomatology course (see Fig 6) is understandable, as Dentistry is a stand-alone program of study in the Czech Republic, and students in General Medicine are merely introduced to this subject. Ophthalmology does not occupy large space within the medical curriculum and that it also has a low perceived importance. In fact, the distant location of preclinical practice in the course of Internal Medicine can be justified with regards to the involvement of different disciplines of internal medicine; otherwise, a high consistency of these disciplines (i.e., Diabetology, Nephrology, and Gastroenterology in the center of the schema [light red color]) is evident. A similar consistency can be seen in the location of courses focusing on community care and public health, including Clinical Practice in Community Medicine (marked in yellow). A high degree of content similarity (according to the location) can also be seen in the preclinical courses (i.e., Basics of Medical Terminology, Anatomy 1, 2, and 3), with an overlap with Histology and relationships with other clinical courses (marked in light red). The distant location of the Imaging Methods course is troubling, in terms of the curriculum consistency. On the one hand, one can argue that imaging techniques in this LU are rather specific, in that it teaches about uncommon examinations, as the common diagnostic methods are shown in individual specialized courses. On the other hand, relative serious shortcoming of this course’s remoteness is that it has no interconnection with other clinical disciplines; each clinical field/course/LU must certainly mention some examination techniques including imaging techniques, as mentioned above, and therefore it is essential to respect both horizontal and vertical interdisciplinary connections and relationships, which are based on a mutual sharing of requirements by individual teachers. The evaluation is also essential for the identification of the extent to which knowledge and skills have been acquired in individual courses. The general view of the curriculum is consistent, involving sporadic distant points that represent courses that focus on soft skills (i.e., Ethics, Communication, and Psychology) on the one hand, and courses that focus on specialized fields of medicine (i.e., Stomatology, Ophthalmology) on the other hand.

### 4.2 Limitations

Regarding medical curriculum innovations, we are investigating how to further optimize our curriculum innovations platform towards interoperability powered by broadly accepted standards and recommendations. The MedBiquitous organization has developed American National Standards Institute accredited technical standards for the health professions education (http://www.medbiq.org/), and for different purposes such as measuring students’ performance, describing health competency frameworks, learning activities and content, and others. In the near future, we will adopt and implement two MedBiquitous standards, Competency Framework and Curriculum Inventory Standard [40] into our curriculum management system. The former allows LO to be used as the backbone of curriculum management systems, and enables users to search for resources that address a specific competency and determine where competencies are addressed in the curriculum. The latter is used to define curriculum data within a specific health profession education program, to facilitate their exchange, and to shift curriculum mapping and reporting from a disjointed and institution-specific undertaking to something that is shared among multiple medical schools and across whole medical education systems. We aim to be able systematically construct the complete profile of a graduate, which will be based on all the available descriptive metadata attributes. The broad support for the mentioned standards gives us a challenging opportunity to determine how to compare various characteristics of students across standardized curriculum management systems.

### 4.3 Comparison with prior work

Prior published works have introduced powerful existing virtual learning environments that focused on the curriculum management and relating planning activities but only from a certain perspective. Usually, these systems generate a huge amount of data, but they unfortunately differ in their levels of detail and their description style. Logically, a lack of any kind of standardization, unification, or common parameterization hampers global transparency and comprehensibility. As a result, it is very difficult to view the whole study field from a broader perspective, and diminishes the possibility of searching easily across the curriculum. Therefore, we decided to build a platform that covers all elements associated with global curriculum harmonization, including a detailed parametric description down to the level of the LUs and LOs. Thanks to this platform, we were able to classify learning activities appearing in the medical curriculum, in accordance with a specialized biomedical dictionary that took the form of aggregated tabular and graphic outputs, including interactive visualizations [41]. For further progress in in-depth curriculum analysis and advanced reporting, this unification on standards level is an essential step forward.

## Conclusions

An original method of fostering curriculum innovations and harmonization was introduced in this paper. The presented solution can be used for the potentially perpetual process of specification and subsequent upgrades in a curriculum at a higher educational institution, and can provide tools to describe the educational process as effectively and easily as possible. Our endeavor was to describe a solution to curriculum mapping issues, and to show how it might make a particular study program more transparent for a broad academic community. In comparison with previously published approaches, we present a comprehensive system that includes a proven planning model based on the outcomes-oriented paradigm, which was adopted in practice by hundreds of senior curriculum designers and innovators as well as by thousands of students in the general medicine study program at Masaryk University. Furthermore, a pilot exploration of a medical curriculum that used social network analysis was performed, and outlying and overlapping medical disciplines were identified.

Regarding reproducibility, the implementation of the presented solution is fully independent of the particular information and communication technologies, as well as the particular field of study to be innovated and harmonized. The basic preconditions for running similar projects in other faculty are: (i) definition of curriculum in accordance with a proposed planning model; (ii) use of a pre-defined parametric structure of the study fields, providing an easy way how to extract required information from database; (iii) engagement of enthusiastic and reliable authoring and reviewing teams of curriculum experts; and (iv) specification of particular curriculum mapping issues, which identify information rich data relations and offer a clear and transparent overview, and in turn creates simpler understandings of the curriculum structure. This paper is oriented to a community interested in a curriculum harmonization process provided by HEIs, especially teachers, tutors, curriculum designers, institutional management, and anyone who might be engaged in the innovation of educational processes. The target group of the proposed platform includes the broader academic community: students, teachers, and the management of HEIs. In our future work, we will initiate the innovation of e-learning content linked to the LUs that have already been restructured by the curriculum designers/innovators. The methodical development and careful implementation of our solution integrates state of the art technologies for effective data handling. Moreover, it establishes a structured environment for further innovation, such as another module (e.g., the reporting tools, which will be developed), in order to provide a real time visual analytics service. We investigate how this tool could both represent the complex curriculum to enable easier analysis and processing, and also elicit scientific value currently hidden in curricula data.
